# Public support for European cooperation in the procurement, stockpiling and distribution of medicines

**DOI:** 10.1093/eurpub/ckaa201

**Published:** 2021-01-17

**Authors:** Roel Beetsma, Brian Burgoon, Francesco Nicoli, Anniek de Ruijter, Frank Vandenbroucke

**Affiliations:** 1 Amsterdam Centre of European Studies, University of Amsterdam, Amsterdam, Netherlands; 2 Faculty of Economics and Business, University of Amsterdam, Amsterdam, Netherlands; 3 Faculty of Social Science and Behavior, University of Amsterdam, Amsterdam, Netherlands; 4 Faculty of Political Economy, University of Ghent, Ghent, Belgium; 5 Amsterdam Centre for European Law and Governance, Faculty of Law, University of Amsterdam, Amsterdam, Netherlands; 6 University of Amsterdam, Amsterdam, Netherlands

## Abstract

**Background:**

The COVID-19 outbreak has heightened ongoing political debate about the international joint procurement of medicines and medical countermeasures. The European Union (EU) has developed what remains largely contractual and decentralized international procurement cooperation. The corona crisis has broadened and deepened public debate on such cooperation, in particular on the scope of cooperation, solidarity in the allocation of such cooperation, and delegation of cooperative decision-making. Crucial to political debate about these issues are public attitudes that constrain and undergird international cooperation.

**Methods:**

Our survey includes a randomized survey experiment (conjoint analysis) on a representative sample in five European countries in March 2020, informed by legal and policy debate on medical cooperation. Respondents choose and rate policy packages containing randomized mixes of policy attributes with respect to the scope of medicines covered, the solidarity in conferring priority access and the level of delegation.

**Results:**

In all country populations surveyed, the experiment reveals considerable popular support for European cooperation. Significant majorities preferred cooperation packages with greater rather than less scope of medicines regulated; with priority given to most in-need countries; and with delegation to EU-level rather than national expertise.

**Conclusion:**

Joint procurement raises delicate questions with regard to its scope, the inclusion of cross-border solidarity and the delegation of decision-making, that explain reluctance toward joint procurement among political decision-makers. This research shows that there is considerable public support across different countries in favor of centralization, i.e. a large scope and solidarity in the allocation and delegation of decision-making.

## Introduction

The outbreak of COVID-19 highlights a crucial challenge for international cooperation regarding the availability of medicines. On the one hand, the crisis underscores the need for states to collaborate in the procurement, allocation and stockpiling of medical countermeasures, particularly in a health emergency.^1^^,^^2^ On the other hand, the crisis awakens protectionism and a retreat to national sovereignty in public health. These offsetting responses play out with respect to key aspects of supranational collaboration in public health, including: scope (to what extent should such collaboration be limited to very specific situations?), solidarity (can priority support be given to countries most in need?) and delegation (can governance be delegated to supranational institutions and expert bodies?).

These features of international collaboration are debated political choices, worldwide and among the Member States of the European Union (EU). The COVID-19 crisis has raised the political sensitivity of such choices, making citizens’ support (or opposition) a crucial policy factor for any given step toward (or away from) supranational collaboration. Despite the salience and importance of citizen support, we have little systematic information about citizen attitudes toward supranational cooperation on medical procurement, and none on the attitudes toward the particular issues of scope, solidarity and delegation.

### Research in context

There has been no research measuring population support for interstate solidarity in joint procurement of medical countermeasures to diseases. The WHO’s latest 2016 literature review on research into public procurement practices, including research into initiatives in regions other than the EU, highlights findings that success of initiatives depended on political trust in the process and in transnational cooperation, and an international organization leading and running the collaborative procurement (see supra note 1, WHO 2016). That research was based on analysis of policy documents, qualitative interviews and survey research of experts and civil servants.

Population support as a precursor for political trust and feasibility, hence, is a critical success factor for interstate joint procurement. Earlier research into the EU’s handling of the Swine flu outbreak taught us that political buy-in for cross-border solidarity amidst a crisis is difficult to organize. Such buy-in concerns both citizen support and policymakers. While citizen support was not researched, qualitative assessment indicated that national policymakers supported EU solidarity neither before nor during the crisis. Yet, afterwards they demonstrated more willingness to organize solidarity by approving the legal basis for the ‘Joint Procurement Agreement’ (JPA).^3^ This highlights anew the issue of citizen support.

### Added value of this study

We report results from a recent five-country randomized survey experiment into public attitudes toward joint procurement and allocation of medical countermeasures in the EU. Despite some differences across sample countries, our results reveal substantial support to jointly procure medical countermeasures, not only in health emergencies. Support is greater for joint measures covering more rather than a limited set of medicines, for granting access to countermeasures based on medical need (solidarity), and for delegating to European expertise to administer joint measures. This study provides evidence of population support for a more central EU-role, including an extension of the existing practice toward redistribution based on medical need. It also signals the possibility of enlarging this cooperation from cross-border threats toward medicines more generally, organizing health solidarity in international settings.

## The EU context and political debate

The most far-reaching international joint procurement scheme, and a frontrunner example in the COVID 19 outbreak, is that of the EU. Whereas other schemes might be limited to information-sharing or price-sharing, the EU’s regime for joint procurement opens the door to interstate solidarity through redistribution of medical countermeasures.^4^ However, it is predominantly a voluntary and contractual, rather than a supranationally-binding, scheme of cooperation.

The European Commission had been encouraging Member States to agree to a joint procurement scheme since the SARS and Avian Influenza outbreaks, but only after the influenza AH1N1 outbreak Member States accepted a legal basis for the 2013 JPA.^5^ This basis limits the scheme’s scope to cross-border health emergencies (Decision No.1082/2013/EU). The JPA applies to joint procurement of medicines (antivirals, treatments or vaccines), medical devices (infusion pumps, needles) and ‘other services and goods’ that mitigate or respond to cross-border threats to health, including laboratory tests, diagnostic tools, decontamination products, masks and personal protective equipment.^6^

The JPA is based on voluntary participation of Member States, and has a largely contractual, ‘intergovernmental’ character. The Commission plays a central organizational role and heads the administrative meetings. But for each procurement, a separate committee of representatives of participating Member States is to be established. With each tender, participating states decide on criteria governing allocation of medical countermeasures.^7^ Participating states receive the exact amounts they have ordered, but the rate of delivery varies in accordance with allocation criteria (Article 17 (1) EU-JPA). In urgent situations, states may request derogation from these generally applicable criteria, so a state in need may receive the countermeasures at a faster rate than other states (Article 17 (2) EU-JPA). This ‘urgency’ is determined by the Commission and participating states (sometimes through qualified majority vote) based on choices made in advance defining the procurement procedure.

This intergovernmental character of the EU’s joint procurement is not happenstance: any development of EU cooperation in public health and cross-border health care must overcome significant political reluctance.^8^Azzopardi-Muscat *et al.* (2017, pp.55–56) emphasize that the voluntary nature of the JPA and its ‘ownership’ by participating Member States were preconditions for its acceptance and development. Simultaneously, these scholars explain how this set-up comes with limitations: the incentives for Member States to participate differ, and securing their ongoing commitment is difficult (for instance, they can organize parallel national procurement for the same products); and representation in the scheme, and a Member State’s voting weight is proportionate to its ‘buy-in’ or investment in the particular procurement, hampering the position of smaller states that likely have most to gain from the scheme .

Since the outbreak of COVID-19, the European Commission has used the existing framework as expediently as possible to enhance Member States’ ability to assemble medical countermeasures. However, the outbreak also highlighted inefficiencies, due to the predominantly intergovernmental and voluntary process and the legally embedded possibilities for non-solidary behavior by individual states. In June 2020, the EU adopted a Vaccines Strategy, which uses the emergency procurement regime ‘rescEU’ based on the EU’s powers in the field of civil protection.^9^ This created an ad hoc and temporary centralized procurement capacity, using JPA legal procedures and infrastructure in conjunction with the rescEU emergency regime to make pre-purchase agreements with the 2.3 billion euro emergency fund and vaccine developers for the whole EU. These ad hoc arrangements underscore the importance of a political discussion on a permanent central capacity for the procurement of medical countermeasures and their distribution in accordance with need.

The arguments for such a central capacity are three-fold. First, central procurement makes it harder for pharmaceutical companies to play-off Member States against each other by threatening not to supply to an individual state trying to negotiate lower prices. Second, having a common stockpile of medical countermeasures promises efficiencies by mitigating excess demand in some countries and excess supply in others. Third, because the common stockpile is larger than any potential national stockpile, it has greater firepower to target outbreaks of infectious diseases wherever and as soon they emerge. These points imply that without rules and procedures set *ex ante* and without credible commitment to those rules by all Member States, individual countries will be tempted to secure as much medicine supply as possible at the cost of other countries.^10–13^

Such centralization is subject to considerable debate in the EU and its Member States. Citizen support is crucial to any effort toward more centralization in democratic polities.^14^ The issue is not only whether populations support some supranational-EU provisions, but also what kind of provisions are favored with respect to the scope of medicines covered, the solidarity in allocation, and the delegation to national or EU expertise.

In March 2020, we conducted a survey experiment in five EU Member States to gauge citizens’ attitudes toward the joint procurement of medicines, focusing on these dimensions of ‘scope’, ‘solidarity’ and ‘delegation in supranational expertise’. Our experiment sheds light on the feasibility of cross-border solidarity in public health policies. Solidarity is key to risk sharing and redistribution in the realization of universal health care ‘within’ states, but also in relationships with other states in public health policies.^15^^,^^16^

## Methods

Our research design is based on a conjoint experiment,^17–20^ fielded among 10 000 respondents in France, Germany, Italy, the Netherlands and Spain. These countries vary with respect to healthcare systems and economic performance, and capture a balance of northern and southern European polities that may differ in views on EU healthcare solidarity. Fieldwork was conducted in March 2020 by the survey company IPSOS by means of their online panels, from which a sample of 2000 individuals was drawn in each of the five countries studied.

The internal validity of the experiment rests on the random assignment of the treatments to the respondents that allows a causal interpretation of the responses to differences in the treatments along the three dimensions. We also control for individual-level attentiveness through an attention check built into the survey questionnaire, and our findings are robust to dropping respondents who provide inconsistent answers.

This being an original experiment, we cannot directly compare our results to independent studies. But the external validity is enhanced by the sample’s representativeness relative to the population, reflecting the quality of the panel and the use of country-specific sample quotas for age, gender, education, occupation and regional distribution. Furthermore, our trust in external validity is helped by the high correlation (0.87) between the level of support for EU membership found in our survey and that found in an identically worded question from a November 2018 survey in the same countries.^21^

Respondents were asked to choose among and judge different policy packages, containing hypothetical procurement and allocation policies. These questions were introduced by a general framing on medical procurement in Europe (see Supplementary Appendix S1). We then presented respondents with policy packages varying across three dimensions of their design. The first dimension (question) covered the ‘scope’: ‘For which medicines should collective EU purchases be organized?’ (answers: either ‘Only for a limited set of medicines used to stop large-scale disease outbreaks’; or ‘For all medicines where collective purchase can be financially beneficial’). The second dimension referred to the presence or absence of need-based ‘solidarity’: ‘Is there priority access to medicines?’ (answers: either ‘No, each participating country has its own fixed share, which it can use whenever and how it wants’; or ‘Yes, in order to prevent a contagious disease from spreading, some countries may have priority access to the common stockpile’). The third dimension was about ‘delegation’ to international institutions and expertise: ‘Who decides about the way in which the medicines are to be used?’ (answers: either ‘Experts in a common European agency’; or ‘Experts in national agencies decide’) (see Supplementary Appendix S1). Respondents were asked to assess three pairs of packages by indicating which one of the packages in each pair they prefer (or least object to), and also by rating each of the six observed packages individually as something they either reject or support.

This results in two measures of how much respondents embrace or eschew a specific procurement policy. *Choose Package* is based on whether or not a given package-pairing-respondent-country was chosen (0 = judged as worse than the paired alternative; 1 = judged as better than the shown alternative), while *Support Package* is based on the (five-point Likert scale) rating that respondents gave to each of the six packages they saw (regardless of choice per pairing): 1 = strongly against; 2 = somewhat against; 3 = neither in favor or against; 4 = somewhat in favor; 5 = strongly in favor. The *Choose Package* and judgment-based *Support Package* correlate highly (Pearson’s correlation coefficient = 0.51). Our analysis accounts for differences between countries and possible sampling composition-effects (controlling for age, gender, income, education, pairing order of questions, worry about COVID-19, etc.), and also seeks to ensure that our respondents understood the questions by focusing on those who passed a standard attention check (weeding-out 14.7% of the sample).

## Results

**Figure 1:**
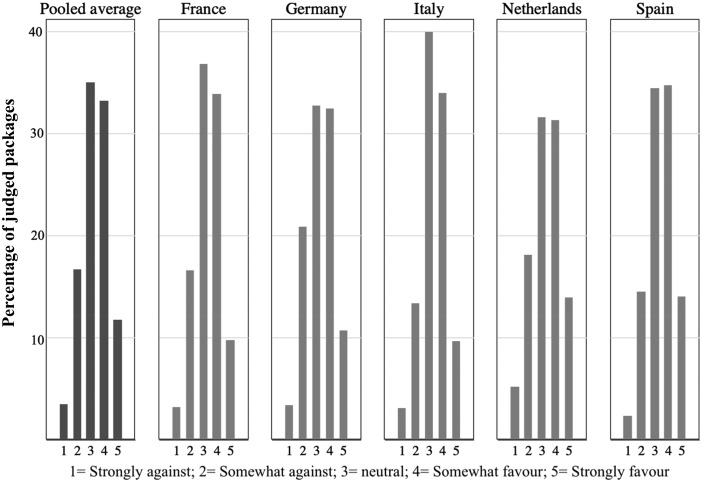
Support and opposition to EU procurement, pooled and by country. Share of total sample judgments of reviewed packages: strongly against (1); somewhat against (2); neither against nor favor (3); somewhat favor (4) and strongly favor (5).

Both *Choose Package* and *Support Package* provide bases for judging the policy ingredients that respondents embrace or eschew. The *Support Package* measure, however, also provides a basis for gauging overall support for EU joint procurement generally, since a judged package’s policy features were randomized and since respondents can express opposition or neutrality as well as support for a package. Figure 1 provides a snapshot of such support—averaged across all possible policy combinations shown (randomly) to our respondents. It displays the share of packages toward which respondents were strongly against (1), somewhat against (2), neither against nor favor (3), somewhat favor (4) and strongly favor (5). The figure presents results for the pooled sample and then for each of the five national sub-samples.

For both the pooled sample and national sub-samples, we see sizeable support for joint procurement policy: for the pooled sample (44.7% somewhat or strongly favor, vs. 19.5% somewhat or strongly against); for France (43.6% favor, 19.9% against); for Germany (43% favor, 24.1% against); for Italy (43.8% favor, 16.5% against); for Netherlands (45.3% favor, 23.4% against) and for Spain (49% favor, 16.9% against). Moreover, the support is balanced over the sample countries, despite the frequently reported differences in politicians’ willingness to transfer national competences to the EU.

Conjoint analysis is particularly useful to assess the impact of specific design features of the policy packages on support or opposition. This is possible by regressing support or choice of a package on the (randomly assigned) policy features across the dimensions. Our baseline estimates focus on *Support Package (binary)* (0 = strongly or somewhat against, or neutral; 1 = somewhat or strongly favor), and on our key explanatory factors: dummies for each value of each dimension. Being experimentally derived, these dimension attributes can be seen as empirically orthogonal to one another (see supra note 17, Hainmueller et al.),^22^ such that the marginal effects of policy attributes can be estimated by linear OLS regression with robust standard errors clustered by respondent, regressing support or choice on dummies for each level of each attribute (excluding a reference category for each dimension). Our preferred specifications include individual-level controls for age, gender, income, education, COVID-19 worry and a package’s pairing-order. These controls address possible remaining omitted variable bias for individual conditions relevant to international solidarity,^23^ and mitigate heteroskedasticity and composition-effects or effects due to the order in which packages are shown to the respondent. However, the baseline results are robust to alternative specifications in terms of whether we focus on *Choose Package* or categorical measures of *Support Package,* on other respondent or package controls, or different estimators.

**Figure 2:**
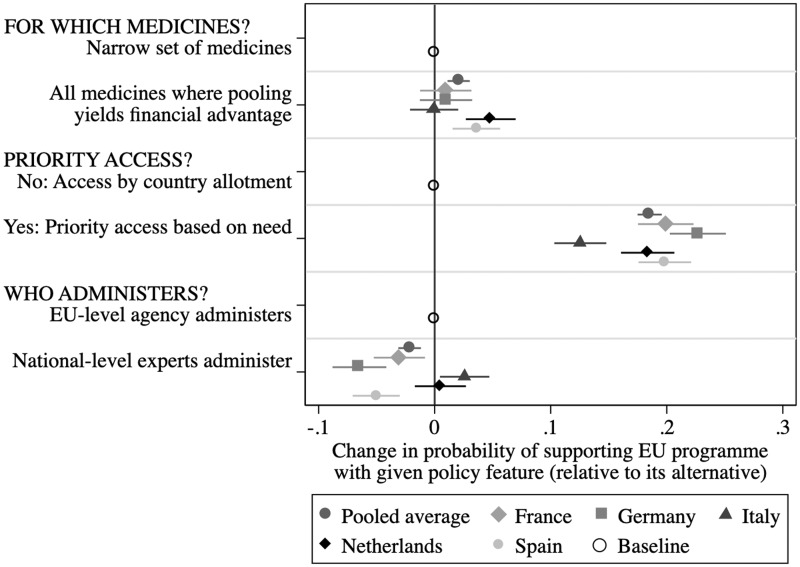
Average marginal component effect of policy attributes on *Support Package*, pooled average and by sub-sample country

Figure 2 summarizes the key results, focusing on the ‘average marginal component effect’ (AMCE) of a given value of each of the dimensions. Such AMCEs gauge the causal effect of a given attribute value, compared to the baseline attribute value, on the probability of supporting a package. The AMCE for each attribute level is equal to the estimated coefficient on its dummy. The relative size of how much a given policy feature influences a respondent’s support for EU policy must be judged in reference to the horizontal axis capturing the support level at a given dimension’s baseline value (see the hollow circles on the line). We also show the 95% confidence intervals of these effects. The figure displays the results for the pooled average of all respondents (given by the first dark dots) and for the country sub-samples.

The patterns include meaningful differences across the countries but are consistent in terms of what most drives support. First, a respondent’s judgment of packages tends not to be strongly driven by whether the policy covers limited or all relevant medicines. Public support increases with (a significant) 3 percentage points if the policy includes such coverage, compared to a policy focusing only on a narrow set of medicines (the baseline). This pattern, however, masks differences in degree between countries, with only respondents in the Netherlands and Spain being statistically so inclined (the other polities not significantly more or less likely to support procurement covering more medicines).

Second, respondents are substantively and statistically significantly more likely to support giving priority access to particular countries to prevent contagion: focusing on ‘PRIORITY ACCESS’, we see that support tends to be about 19 percentage points higher when a policy gives priority access to countries where a contagion can be traced, i.e. based on need, as compared to merely providing access based on pre-set and fixed country quotas (the baseline). All country sub-samples undergird this pattern, though to different degrees (Italians are ‘only’ 12% more likely to support such priority access, while Germans are more than 23% more likely to do so).

Third, we see meaningful differences across countries in how support for joint procurement is shaped by EU-level vs. national-level experts administering of the policy. The pooled average suggests that respondents are weakly disinclined (about 2% less) to support national-level administration compared to EU-level administration (the baseline). But this pro-EU-level pooled result masks differences across sub-samples: French, German and Spanish respondents drive that result, while the Italians tend to be more positively swayed by national-level administration and the Dutch are not swayed one way or another by national versus EU-level control.

**Figure 3:**
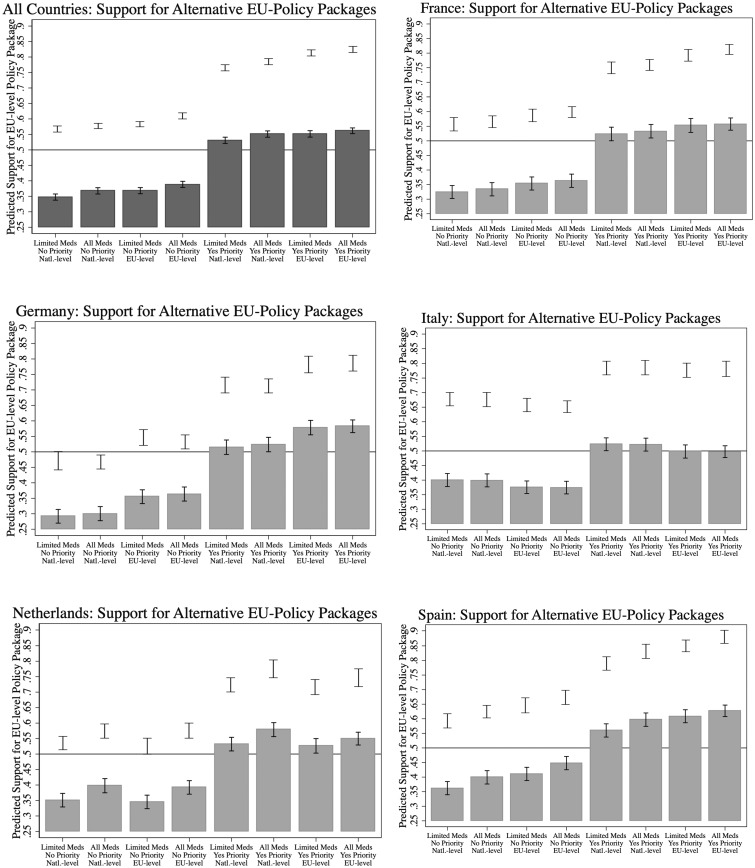
Predicted support for possible policy packages, pooled and by country. Bars indicate predicted support based on *Support EU Procurement* (1 = somewhat or strong support; 0 = neutral or somewhat or strong opposition); upper intervals indicate predict *Support-vs.-Oppose EU Procurement* (1 = somewhat or strong support; 0 = somewhat or strong opposition)

How such marginal effects play out for predicted support for EU procurement is captured by figure 3, modeling the (counterfactual) support for each of the eight possible combinations of alternative policy attributes. We include a low-floor and a high-ceiling estimate. The low-floor is based on results from *Support Package* commanded by a given package, shown by gray bars with 95% confidence intervals. This low-floor estimate presumes that, in an imaginary referendum, all neutrals would vote against the package. More likely is that at least some of the neutrals would support the package or not participate in a vote. Hence, we also show a ‘high-ceiling’ estimate that presumes that neutrals stay home on election day. These high-ceiling predictions are captured by the vertical solid-line 95%-confidence intervals of predicted support. Figure 3 shows the results, again for the pooled sample and the five country sub-samples.

In the pooled sample, the most supported package features a combination where the degree of policy centralization is at its maximum, i.e. joint procurement of all medicines where there is a benefit, allocation based on urgency and execution at the EU-level. Also noteworthy, and in line with the marginal effects summarized in figure 2, the priority access dimension is what most makes or breaks support for EU procurement: “urgency based” versus “contribution based” appears to be the significantly most favored, and what pushes even the low-floor results above the 50% support threshold (see upper-left panel, the last four packages). The other conditions do not have much effect. The country-specific results broadly follow this pooled pattern. For each country and both support measures, all four policy packages featuring priority access lie uniformly above all four packages featuring access based on contribution. Noteworthy deviations from the pooled results, however, are found for the Netherlands and Italy, where packages with national-level governance are most prized over those with EU-level governance.

To what extent did the timing of the survey—during the global Covid-19 outbreak—influence respondents’ views? We can isolate the specific effect of the Covid-19 outbreak by comparing the Dutch results of our main survey (whose fieldwork took place in the last week of March 2020) with the results of a pilot study, fielded in the Netherlands in November 2019, ‘before’ the Covid-19 outbreak. Results are stable, both in overall level of support and in the dimension-specific effects (available on request).

The main survey took place when the EU response to the pandemic was salient (March 2020), but politicization of the issue was yet to happen, at least to the extent observed in June 2020. While this unique timing gives us a glimpse of what ‘pristine’ public opinion may look like for salient but non-politicized issues, our results must be interpreted with caution, as they do not automatically ensure that stable majorities for an EU layer of health policy as envisaged in the experiment would survive a politicization, e.g. as bitter as that observed during the discussion of the EU Recovery Fund in late Spring 2020. While we present preliminary evidence of support for centralization, only a repetition of the experiment can ultimately confirm the stability of such evidence.

## Conclusion

Effective international joint procurement requires sufficient scope, capacity to organize solidarity with countries that are most in need, and delegation in the decision-making. Whether populations support such centralization of procurement and its extensions into distributional choices and pooling of expertise is a crucial question for health governance in Europe. A survey experiment in five countries shows not only potential majority support for EU joint procurement; results also suggest that the most supported package features a combination where the degree of centralization is at its maximum, i.e. joint procurement of ‘all’ medicines where there is a benefit to be reaped, allocation based on urgency and execution at the EU-level.

## Supplementary data

Supplementary data are available at *EURPUB* online.

## Funding

The funding for this research was provided by the University of Amsterdam through the Research Priority Area for European Studies and the Amsterdam Centre for European Studies.

*Conflicts of interest:* None declared.

**Key points**

- EU joint procurement raises delicate questions with regard to its scope, the inclusion of cross-border solidarity and the delegation of decision-making, which explains reluctance toward such aspects of joint procurement among political decision-makers.
- Crucial to political debate about these issues are public attitudes that constrain and undergird international cooperation.
- This article reports on a randomized survey experiment among EU citizens regarding EU joint procurement of medical countermeasures to large scale disease outbreaks.
- In all country populations surveyed, the experiment reveals considerable popular support for EU joint procurement, where significant majorities preferred cooperation packages with greater rather than less scope of medicines regulated; with priority given to most in-need countries; and with delegation to EU-level rather than national expertise.
- This research shows that there is a considerable public support across different countries in favor of centralization, i.e. a large scope, solidarity in the allocation and delegation of decision-making.

## Supplementary Material

ckaa201_Supplementary_Data
