# Supplementary material for: Public support for European cooperation in the procurement, stockpiling and distribution of medicines
Source: Eur J Public Health. 2021 Jan 17;31(2):253–8. doi: 10.1093/eurpub/ckaa201 (PMC7928975; doi:10.1093/eurpub/ckaa201)
Supplement: ckaa201_Supplementary_Data [file ckaa201_supplementary_data.zip › ejph-2020-07-om-0795-File006.rtf]

Methodological appendix XXX1.	Survey design.The survey has been administered online to a representative sample of 2000 individuals in each of 5 countries (total sample 10,000 respondents). Countries were selected for their variation on economic policy, preferences for European integration, social welfare model, institutional set-up.The survey was administered by the specialized survey company IPSOS to randomly drawn members of their online panels. To ensure (even) higher representativeness of our sample beyond  standard IPSOS surveys, we requested sampling quotas for education (3 categories), equivalized household income (3 categories), regional distribution (NUTS1), sex (2 categories), age (3 categories) and profession (10 categories). The delivered sample matches all categories with a discrepancy from the actual population below 3%, with exception of somewhat larger discrepancies for professions in some countries and for top earners for equivalized household income. The survey contains 2 separate survey experiments, a battery of control questions, and an attention check designed to identify careless or inattentive respondents.2.	Experimental design.The survey included 2 separate experiments. This article focuses on the second experiment. Both experiments are “conjoint experiments”, whereby individuals are presented with two randomly sorted policy packages, varying across a number of dimensions, side by side. The values of the dimensions are randomized and constitute the treatments of the experiment. For each pair, they first indicate which alternative package they prefer, and then they rate each package independently on a 5 points scale from “very much against” to “very much in favour”.The experiment is repeated 3 times, such that every respondent is confronted with 6 packages, has to rate 6 packages, and has to choose one in each of the three pairings of packages. Since the dimensions are randomly combined into packages, the effect of such treatments on the dependent variables of choice can be interpreted causally. The leading hypotheses driving survey design have been pre-registered with a survey-experiment pre-registration authority, and these are available in anonymized format upon request to the editors of the present journal.3.	Pre-analysis data transformations.Data are collected at the level of the individual; every individual is  confronted with 6 packages, and asked to choose 3 (as preferred or least opposed) and rate all 6 packages regardless of such choice.However, the analysis takes place at the level of package, so to discern the effect of a package having certain randomly sorted features on the support expressed for that package, or on the probability of it being chosen or not chosen. Hence, data are transformed from a “short” format where individuals are the unit of observation, to a format where the package is the unit of observation. Since every respondent is confronted with 6 packages, the actual number of observations for the models in this study can go up to 60.000. We account for the fact that multiple evaluations are attributable to the same respondents in multiple ways. For instance, models 1-5 in table A1 cluster the standard errors at individual-respondent level, while model 6 uses individual-respondent fixed effects. The study uses two main dependent variables: package choice and package support. Package choice is naturally a binary variable, since every package can either be chosen or not be chosen. Package support instead is originally casted as a score variable on a 5-points scale from “very much against” to “very much in favour”. To simplify interpretation, we adopt a binary transformation of this score variable, whereby negative and neutral assessments (values 1-3, respectively) are recoded as 0, and positive assessments (values 4-5, respectively) are recoded as 1. 4.	Model specifications.We look at a very wide range of different model specifications, a representative subset of which is reported in table A1. For all models, we use the estimator that makes the interpretation the easiest, namely OLS. Model 1 and model 2 are different only insofar they look, respectively, at the choice variable and at the support variable. They both estimate the changes in the respective DV attributable to the treatments on the full sample (i.e., inclusive of inattentive individuals). It only includes those features that are directly part of the experiment: the dimensions of the experiment itself (whose coefficients are to be interpreted as changes over the baseline alternative), a control for whether individuals passed the attention check, the pair in which each package was featured (first, second or third repetition of the experiment). We also control for one feature of the other, unrelated experiment (“framing”), which preceded this experiment to make sure than no contamination effects had occurred. Models 3 and 4 repeat the same specification, adding country fixed effects.Model 5 and 6 look at the support dependent variable only. In model 5, we restrict the sample to those respondents who had passed the attention check, and we add an additional set of sociodemographic controls, such as income, age, education, and gender. We also control for individuals concerns for the Covid-19 outbreak, expressed on a scale from 1 to 10. Model 6 is identical to model 5, but uses individual fixed effects, rather than clustering of the standard errors, to account for the fact that multiple observations are cast by the same respondent.Table A1: selected models	model 1. OLS on Choice variable, only experimental variables, full sample, Individuals SE clustering	model 2. OLS on binary support variable (neutrals coded as against), only experimental variables, full sample, Individuals SE clustering	Model 3. As model 1, + country fixed effects	Model 4. As model 2, + country fixed effects	Model 5. OLS on binary support variable (neutrals coded as against), extended controls, attentive respondents subsample, Individuals SE clustering	Model 6. OLS on binary support variable (neutrals coded as against), only experimental variables, attentives subsample, individuals fixed effects	
All medicines 	0.027	0.017	0.027	0.017	0.020	0.021	
	(0.005)***	(0.004)***	(0.005)***	(0.004)***	(0.005)***	(0.004)***	
Priority access	0.227	0.165	0.227	0.165	0.183	0.180	
	(0.005)***	(0.004)***	(0.005)***	(0.004)***	(0.005)***	(0.004)***	
National administration	-0.016	-0.019	-0.016	-0.019	-0.021	-0.022	
	(0.005)***	(0.004)***	(0.005)***	(0.004)***	(0.005)***	(0.004)***	
Second pair	0.001	-0.018	0.001	-0.018	-0.017	-0.016	
	(0.001)	(0.003)***	(0.001)	(0.003)***	(0.003)***	(0.005)***	
Third pair	0.002	-0.021	0.002	-0.021	-0.020	-0.020	
	(0.001)*	(0.003)***	(0.001)*	(0.003)***	(0.003)***	(0.005)***	
Attention Check	-0.000	0.019	-0.001	0.016			
	(0.000)	(0.008)**	(0.000)	(0.008)**			
Economic framing (unrelated)	0.000	0.001	0.000	0.001	0.005		
	(0.000)	(0.005)	(0.000)	(0.005)	(0.006)		
Germany			0.000	-0.011	0.002		
			(0.000)	(0.008)	(0.008)		
Italy			0.000	0.006	0.007		
			(0.000)	(0.008)	(0.009)		
The Netherlands			0.001	0.013	0.024		
			(0.000)	(0.008)*	(0.009)***		
Spain			0.000	0.051	0.060		
			(0.000)	(0.008)***	(0.009)***		
Covid-19: how worried?					0.009		
					(0.002)***		
Female					-0.006		
					(0.006)		
Age					0.018		
					(0.004)***		
Education					0.020		
					(0.004)***		
Income					0.008		
					(0.004)**		
_cons	0.380	0.360	0.380	0.350	0.191	0.372	
	(0.004)***	(0.011)***	(0.004)***	(0.012)***	(0.021)***	(0.005)***	
R2	0.05	0.03	0.05	0.03	0.04	0.04	
N	60,300	60,300	60,300	60,300	44,850	51,444	
* p<0.1; ** p<0.05; *** p<0.01
