# Supplementary figures and images for: Public support for European cooperation in the procurement, stockpiling and distribution of medicines
Source: Eur J Public Health. 2021 Jan 17;31(2):253–8. doi: 10.1093/eurpub/ckaa201 (PMC7928975; doi:10.1093/eurpub/ckaa201)

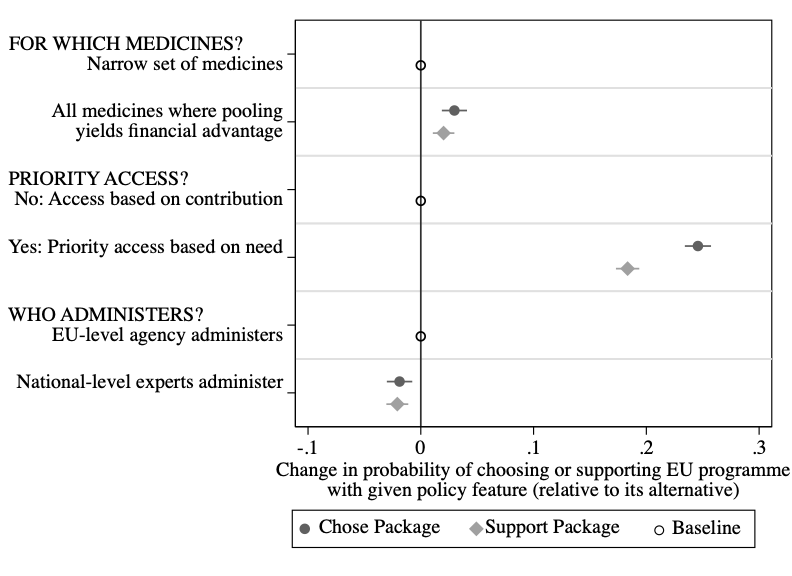

Supplement: ckaa201_Supplementary_Data [file ckaa201_supplementary_data.zip › ejph-2020-07-om-0795-File005.png]
